# Effect of Surface Modifications of Ti_40_Zr_10_Cu_38_Pd_12_ Bulk Metallic Glass and Ti-6Al-4V Alloy on Human Osteoblasts *In Vitro* Biocompatibility

**DOI:** 10.1371/journal.pone.0156644

**Published:** 2016-05-31

**Authors:** Andreu Blanquer, Anna Hynowska, Carme Nogués, Elena Ibáñez, Jordi Sort, Maria Dolors Baró, Berna Özkale, Salvador Pané, Eva Pellicer, Leonardo Barrios

**Affiliations:** 1 Departament de Biologia Cel·lular, Fisiologia i Immunologia, Universitat Autònoma de Barcelona, Edifici Cc, Bellaterra, Spain; 2 Departament de Física, Universitat Autònoma de Barcelona, Edifici Cc, Bellaterra, Spain; 3 Institució Catalana de Recerca i Estudis Avançats, Barcelona, Spain; 4 Multi-Scale Robotics Lab, Institute of Robotics and Intelligent Systems, ETH Zurich, Zurich, Switzerland; Second University of Naples, ITALY

## Abstract

The use of biocompatible materials, including bulk metallic glasses (BMGs), for tissue regeneration and transplantation is increasing. The good mechanical and corrosion properties of Ti_40_Zr_10_Cu_38_Pd_12_ BMG and its previously described biocompatibility makes it a potential candidate for medical applications. However, it is known that surface properties like topography might play an important role in regulating cell adhesion, proliferation and differentiation. Thus, in the present study, Ti_40_Zr_10_Cu_38_Pd_12_ BMG and Ti6-Al-4V alloy were surface-modified electrochemically (nanomesh) or physically (microscratched) to investigate the effect of material topography on human osteoblasts cells (Saos-2) adhesion, proliferation and differentiation. For comparative purposes, the effect of mirror-like polished surfaces was also studied. Electrochemical treatments led to a highly interconnected hierarchical porous structure rich in oxides, which have been described to improve corrosion resistance, whereas microscratched surfaces showed a groove pattern with parallel trenches. Cell viability was higher than 96% for the three topographies tested and for both alloy compositions. In all cases, cells were able to adhere, proliferate and differentiate on the alloys, hence indicating that surface topography plays a minor role on these processes, although a clear cell orientation was observed on microscratched surfaces. Overall, our results provide further evidence that Ti_40_Zr_10_Cu_38_Pd_12_ BMG is an excellent candidate, in the present two topographies, for bone repair purposes.

## Introduction

The use of biocompatible materials for tissue regeneration, tissue engineering and transplantation has increased in recent years. Among different types of biomaterials, metallic alloys are preferred for orthopaedic applications because of their excellent mechanical and physical properties, such as a relatively high elasticity (low Young’s modulus) and hardness. Metallic biomaterials used as permanent surgical implants can be classified into three categories according to their composition: stainless steel, cobalt-chromium and titanium-based alloys [1,2]. The closer the elasticity of the metallic alloy is to that of the bone, the lower is the probability of implant loosening by the stress shielding effect [1]. Ti-based alloys are the most used in medicine for long-term bone implants because their Young’s moduli (55 to 110 GPa) are closer to that of the cortical bone (4 to 30 GPa) in comparison with chromium-cobalt alloys (240 GPa) and stainless steel (210 GPa). Ti-based alloys are used to manufacture hip and knee joints or screws and pins suitable for orthopaedic implants. Among them, Ti-6Al-4V is widely used because of its good mechanical properties, although it has a high Young’s modulus (110 GPa). During the last years, new alloys have been synthesized to improve the elastic properties. Bulk metallic glasses (BMG) are very promising due to their superior strength, relatively low Young’s modulus, high elastic strain limit, excellent corrosion resistance and good wear resistance [3]. Nevertheless, before these new alloys can be applied, their possible toxicity and/or allergenic effects have to be studied. The Ti-Zr-Cu-Pd alloys emerged as a new family of Ti-based metallic glasses in 2007, completely free from toxic elements (like Be or Ni), as an alternative to the previously investigated Ti-Cu-Ni-Zr-Be or Ti-Cu-Ni-Zr-Nb(Ta) bulk metallic glasses [4,5]. In terms of physical properties, the Ti–Zr–Cu–Pd bulk metallic glasses exhibit higher strength (almost twice) and lower Young’s modulus than commercial Ti–6Al–4V [6]. The corrosion resistance of these materials is also very high, due to the formation of stable and protective passive films with enhanced Ti and Zr content [7]. Among the different possible stoichiometries, the particular Ti_40_Cu_38_Zr_10_Pd_12_ composition was reported to be among the alloys with highest glass forming ability [5]. Ti_40_Zr_10_ Cu_38_Pd_12_ BMG also shows good mechanical and corrosion properties, it is not cytotoxic for mouse preosteoblasts cultured for 21 days in the presence of the alloy and it does not induce an inflammatory response by macrophages, making it a potential candidate for orthopaedic implants [8]. In addition, several authors have reported that Cu is an efficient alloying element to develop antibacterial Ti-based alloys, presenting good results against *Salmonella enteritidis* and *Salmonella typhimurium* [9,10].

Besides composition, surface topography of alloys can play an important role on bone integration, including cell adhesion, spreading, migration, proliferation and differentiation [11]. It has been described that mirror-like surfaces show good biocompatibility, allowing osteoblast adhesion, proliferation and differentiation [12,13]. Surface topography can be classified by the scale of the irregularities (roughness) and by their distribution (morphology). According to the scale, macroroughness (>100 μm), microroughness (1 μm-100 μm), submicron roughness (100 nm-1 μm) and nanoroughness (<100 nm) have been defined [14]. It has been described that microrough surfaces allow a better cell proliferation and differentiation than smooth surfaces [15,16]. Moreover, nanorough surfaces allow a better osseointegration and it is believed that irregularities smaller than 100 nm can mimic the nanoarchitecture of natural tissues [17,18]. According to the morphology, although there is not any classification, it has been reported that cells show different responses to different surface morphologies such as grooves [19], tubes [20], meshes [21] or columns [22].

In the present study, we have analysed the biocompatibility of Ti-based alloys as a function of their surface topography. To alter the surface architecture of the alloys, two different approaches were employed: (a) electrochemical surface modification, in which a nanomesh surface is obtained; (b) physical surface modification, in which the surface becomes microscratched. As controls, unmodified mirror-like surfaces were also used. Two alloys were investigated: Ti_40_Zr_10_ Cu_38_Pd_12_, previously reported by our group as a biocompatible material [8], and the commercially available Ti-6Al-4V. The biocompatibility of human osteosarcoma cells cultured on these alloys was assessed by evaluating the cytotoxicity, proliferation, morphology, adhesion, cytoskeleton distribution and differentiation.

## Materials and Methods

### Material synthesis

The master alloy Ti_40_Zr_10_ Cu_38_Pd_12_ (from now on TiZrCuPd) (at.%) was prepared by arc melting a mixture of the highly pure elements (>99,9%) under a Ti-gettered Ar atmosphere. Rods of 3 mm in diameter were obtained from the melt by Cu mold suction casting. Disks of approximately 500 μm in thickness were cut from the as-prepared TiZrCuPd and from commercial Ti-6Al-4V (Alfa Aesar) rods, and carefully grinded with SiC paper (up to 4,000 grit), degreased with acetone and finally cleaned with distilled water in an ultrasonic bath.

### Surface modification

For both compositions (TiZrCuPd and Ti-6Al-4V), two different surface treatments were applied: physical and electrochemical. The physical surface modification consisted of manually scratching mirror-like polished disks along one direction with 1,200 grit SiC paper. A linearly microscratched surface was obtained. The electrochemical surface modification consisted of polarizing the alloys in a 5 M NaOH solution at 25°C. This was carried out in a one-compartment thermostatised three-electrode cell connected to a PGSTAT302N Autolab potentiostat/galvanostat (Ecochemie). An Ag←AgCl (3M KCl) and a Pt wire served as reference and counter electrodes, respectively. Single potentiodynamic polarization scans were run from –0.5 V to +2.0 V at 0.5 mV s^–1^. A fraction of the disks was not subject to any special treatment (mirror-like finish).

### Compositional, morphological and physicochemical characterization

The chemical composition of the electrochemically modified disks was studied by energy dispersive X-ray (EDX) spectroscopy on a Zeiss Merlin field-emission scanning electron microscope (FE-SEM) operated at 15 kV. For this purpose, five disks of each alloy composition were used.

Surface roughness was determined by atomic force microscopy (AFM) using a Dual Scope TMC-26 system (Danish Micro Engineering) working in AC mode. A commercial silicon tip (50–100 KHz resonance frequency) was used to scan two surface areas of 50 x 50 μm^2^ per disk in order to extract the peak-to-valley (PTV) distance and the root-mean-square (RMS) roughness values. One disk was analysed for each surface modification and alloy composition.

Cross-sections of electrochemically modified disks were obtained in a SEM equipped with a focused ion beam (FIB). A gallium ion source with currents in the range 300 pA– 1.5 nA was employed. Samples were sputtered with a thin layer of gold prior to FIB-cutting the area of interest in order to enhance sample conductivity and to prevent any drift or charging. A thin carbon coating was deposited during imaging prior to FIB cross-sectioning to provide smooth cross-sections. Two disks were analysed for each alloy composition.

The wettability of the surfaces resulting from the performed treatments was studied by sessile drop technique. A drop of 1.5 μl of Dulbecco’s modified Eagle medium (DMEM; Invitrogen) was deposited onto the surface of the specimens using a microdispenser and the contact angle was determined with a Contact Angle Measuring System DSA 100 (Krüss) at room temperature (RT). Three measurements of the same disk area were sequentially performed, and two disks were analysed for each surface modification and alloy composition.

### Cell culture

Osteoblast-like Saos-2 (ATCC, HTB-85) cells, derived from a primary human osteosarcoma, were cultured in DMEM with 10% foetal bovine serum (Gibco) under standard conditions (37°C and 5% CO_2_).

### Cell viability assay

Alloy disks were cleaned with absolute ethanol and glued onto glass coverslips with silicone (Bayer), introduced into a 4-multiwell culture plate and sterilized by UV light for at least 2 h. Once sterilized, 50,000 cells were seeded into each well and cultured for 24 h. Cell viability on disk surfaces was evaluated using the Live/Dead Viability/Cytotoxicity kit for mammalian cells (Invitrogen), according to the manufacturer’s protocol. Images from different regions of the alloy disk and from the control culture (without disk) were captured using an Olympus IX71 inverted microscope equipped with epifluorescence. A minimum of 300 cells were analysed per disk for each surface modification and alloy composition. The experiment was performed in triplicate.

### Cell morphology analysis

After the cell viability assay, the same samples were prepared for observation in SEM. Cultured cells were rinsed twice in phosphate buffered saline (PBS), fixed in 4% paraformaldehyde (PFA, Sigma) in PBS for 45 min at RT and rinsed twice in PBS. Cell dehydration was performed in a series of ethanol (50%, 70%, 90% and twice 100%), 7 min each. Finally, samples were dried using hexamethyl disilazane (Electron Microscopy Sciences) for 15 min, mounted on special stubs and analysed using SEM (Zeiss Merlin).

### Cell adhesion and actin cytoskeleton distribution analysis

Cell adhesion onto the alloy surface was analysed using an antibody against vinculin to determine the presence of focal contacts. At the same time, phalloidin was used to visualize actin filaments and their distribution, as reported previously [8]. The same cell culture protocol described for viability studies was employed, but after 24 h of culture the cells were fixed in 4% PFA in PBS for 30 min at RT, permeabilised with 0.1% Triton X-100 (Sigma) in PBS for 15 min and blocked for 25 min with 1% bovine serum albumin (BSA; Sigma) in PBS at RT. Samples were then incubated with 2 μg/ml mouse anti-vinculin primary monoclonal-antibody (Chemicon, MAB3574) for 60 min at RT and washed with 1% BSA-PBS. Then, samples were incubated with a mixture of 1.4 U/ml Alexa fluor 594-conjugated phalloidin (Invitrogen), 6 μg/ml Alexa fluor 488 goat anti-mouse IgG1 and Hoechst 33258 (both from Sigma) for 60 min at RT. Finally, samples were washed in 1% BSA-PBS, air dried and mounted on specific bottom glass dishes (MatTek) using ProLong mounting solution (Life Technologies). Control analyses were performed in absence of the alloy. Sample evaluation was done with a confocal laser scanning microscope (CLSM, Olympus). One disk was analysed for each surface modification and alloy composition.

### Cell proliferation assay

Saos-2 proliferation was determined at 72 h and 7 days using Alamar Blue (Invitrogen). A total amount of 250,000 cells were seeded into each well of a 4-multiwell plate containing the alloy disk. After 24 h, disks with adhered cells on the surface were transferred to a 96-multiwell plate and medium with 10% of Alamar Blue was added into each well and incubated for 4 h at 37°C and 5% CO_2_, protected from direct light. Then, the supernatant was collected and the fluorescence was read using a Cary Eclipse fluorescence spectrophotometer (Agilent Technologies). Cells on the disk were incubated again with fresh medium, and the Alamar Blue analysis was repeated at 72 h and 7 days. All experiments were performed in triplicate for each surface modification and alloy composition. Negative controls without cells were run as well.

### Cell differentiation assay

Differentiation of cells growing on the alloy was evaluated through the alkaline phosphatase (ALP) activity and the detection of calcium deposits, a sign of extracellular matrix (ECM) mineralization. A total amount of 500,000 cells were seeded into 35 mm culture dishes containing an alloy disk and cultured during 14 days, replacing the medium every 3–4 days. All experiments were done in triplicate for each surface modification and alloy composition.

After 14 days in culture, the alloy disk was transferred to an eppendorf tube and the cells were lysed, using CyQuant cell lysis buffer (Invitrogen), for 10 min and vortexed for 15 s. Cell lysates were centrifuged at 12,000 rpm for 4 min at 4°C and the supernatants were used to evaluate ALP activity by quantifying p-nitrophenol, produced by the hydrolysis of p-nitrophenyl phosphate (pNPP). Briefly, 25 μl of 1-step pNPP (ThermoScientific) was added to 25 μl of supernatant. After 30 min incubation at RT, 2M NaOH was added to stop the reaction. The absorbance was measured at 405 nm using a Nanodrop Spectrophotometer (ThermoScientific). ALP activity was normalized to total protein content using the Micro BCA Protein Assay kit (ThermoScientific), according to the manufacturer’s protocol.

Osteoblasts growing on the vicinity of the disks used for ALP activity detection, were stained with Alizarin Red S to detect secreted calcium deposits. After 14 days in culture, cells were fixed in 4% PFA in PBS for 30 min at RT. Then, cells were incubated with 2% Alizarin Red S (Sigma) for 30 min at RT. Finally, samples were washed with milliQ water and visualized using an Olympus IX71 inverted microscope.

### Statistical analysis

Cell viability was analysed using the Fisher’s exact test. Cell proliferation and cell differentiation were analysed using the Kruskal-Wallis test. Statistical significance was considered when p < 0.05. All statistical analyses were performed with the GraphPad PRISM software (6.01).

## Results

### Samples characterization

**Fig 1:**
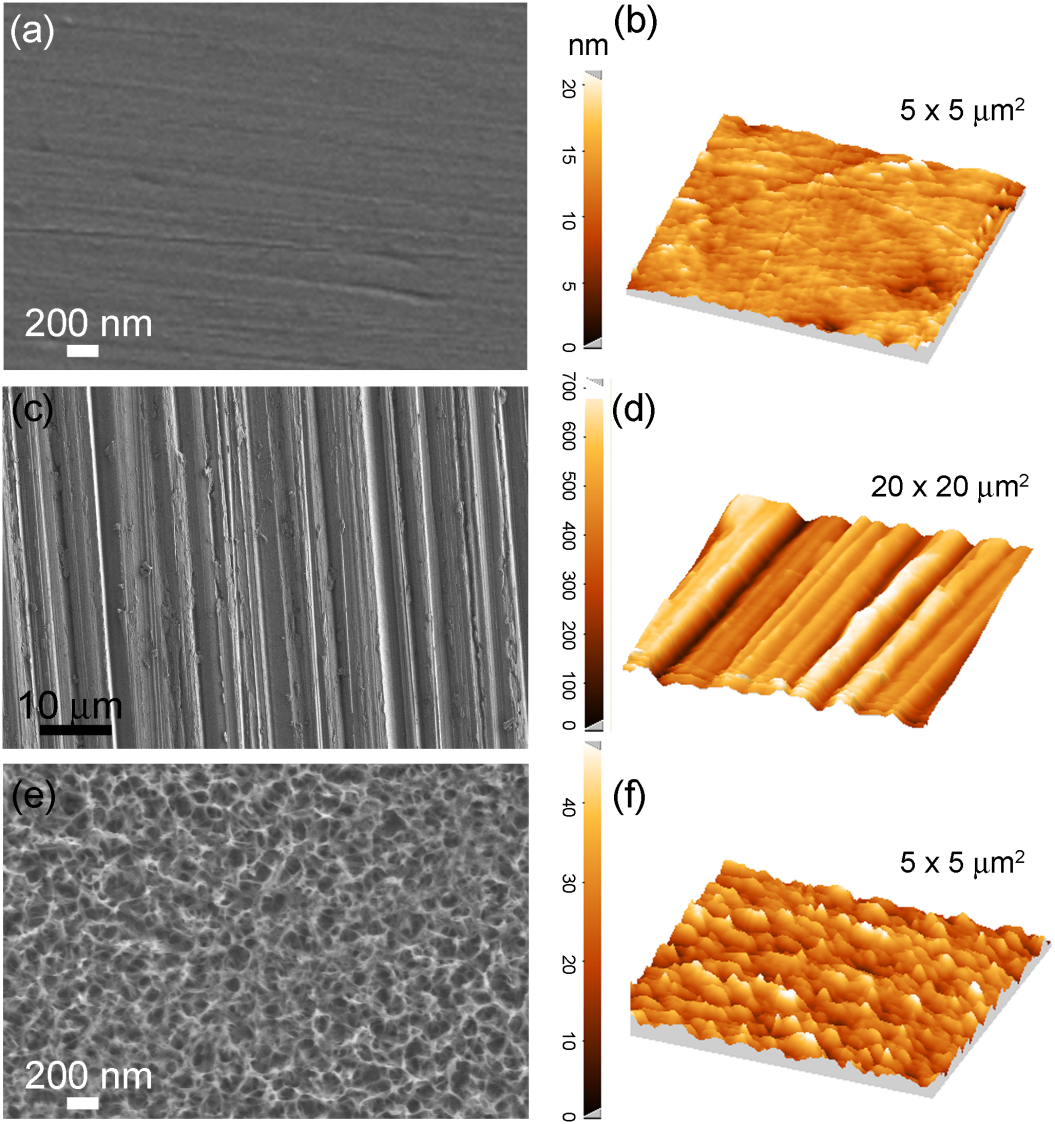
SEM (a, c, e) and AFM (b, d, f) images of mirror-like (a, b), microscratched (c, d) and nanomesh (e, f) surfaces of TiZrCuPd alloy.

**Fig 2:**
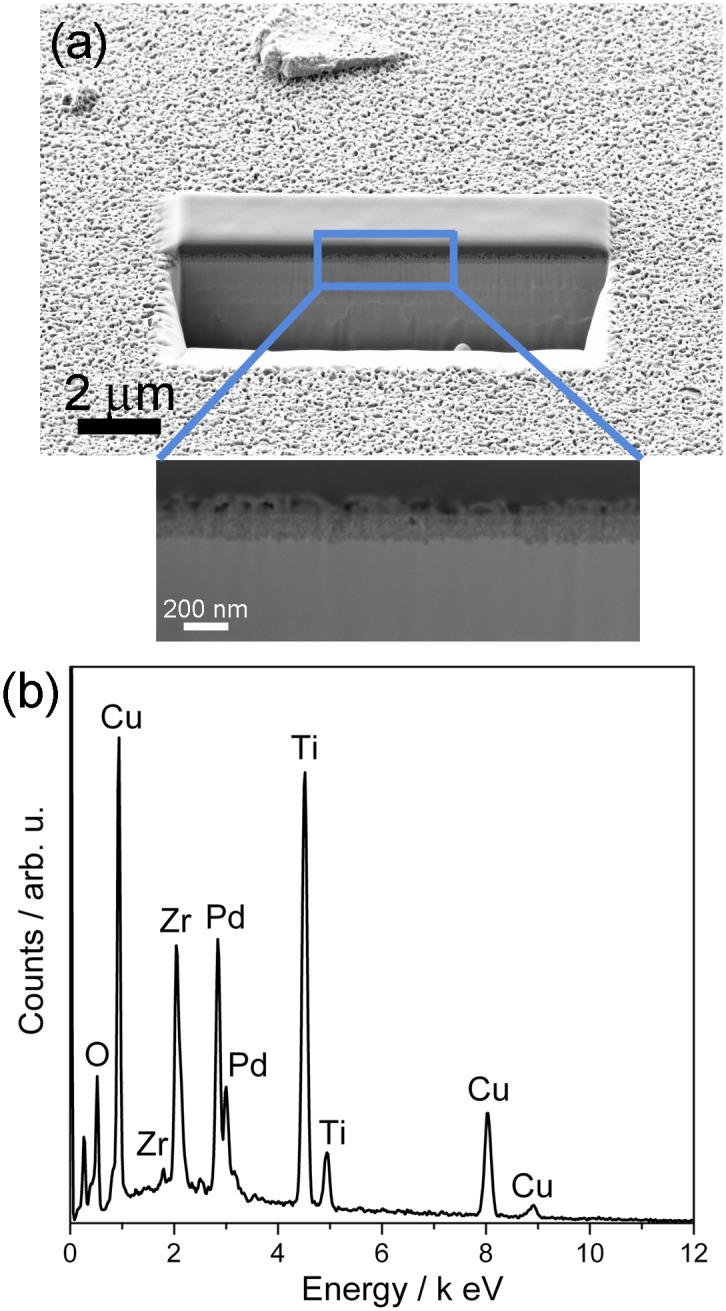
SEM-FIB cross-section (a) and EDX pattern (b) of a nanomesh layer formed by electrochemical treatment of TiZrCuPd alloy.

The physicochemical properties of the surface resulting from the aforementioned treatments applied to TiZrCuPd and Ti-6Al-4V materials were studied by SEM, AFM and contact angle measurements. As expected, the mirror-like polished surfaces were smooth and did not show any relevant feature apart from some residual ultra-fine scratches caused by the polishing (Fig 1(a) and 1(b)). The grooves created during manual scratching of the surface with 1,200 grit SiC paper (microscratched) can be observed in Fig 1(c) and 1(d). Trenches run in parallel over the surface since polishing was conducted in only one direction. Finally, a highly interconnected hierarchical porous structure was observed on the TiZrCuPd surface following electrochemical treatment (nanomesh). The pore walls were very thin, of only a few nanometres in thickness (Fig 1(e) and 1(f)). The same porous structure was observed on Ti-6Al-4V surface (S1 Fig). During potentiodynamic polarization up to 2V, the sample undergoes severe oxidation revealing a nanomesh structure on the surface. A typical potentiodynamic polarization curve is shown in in the supplementary data (S2 Fig). The thickness of this nanomesh layer was around 200 nm, as illustrated in the FIB-SEM cross-section displayed in Fig 2(a). Actually, a magnified detail reveals a two-layer structure; an upper highly porous layer and a more compact layer underneath. Below this 200 nm-thick layer, the alloy remains unaffected. The EDX spectrum of the electrochemically-treated surface is depicted in Fig 2(b). Chemical composition analysis revealed that the nanomesh is made of 27 at% Ti, 7 at% Zr, 21 at% Cu, 9 at% Pd and 36 at% O. This indicates that the network is rich in Ti and Cu oxides. Nevertheless, since the penetration of X-rays in EDX is of the order of a few microns, the elemental composition is likely influenced by the underlying metallic alloy. These results indicate that the applied treatments render surfaces with very distinct features, ranging from micro- to nanoscales. Similar results were observed for Ti-6Al-4V. For this alloy, the nanomesh layer obtained after anodic treatment contains 57 at% Ti, 6 at% Al, 2 at% V and 35 at% O; hence it is mostly made of titanium oxides. Again, taking into account the small thickness of the porous layer and the penetration of X-rays, the percentages are likely influenced by the underlying alloy.

Table 1 lists the RMS roughness (R_q_) and the PTV distance determined by AFM for the different surfaces. These values were obtained on scanned areas of 5 x 5 μm^2^ in the case of the mirror-like finish and the nanomesh surfaces, and larger areas of 50 x 50 μm^2^ in the case of the microscratched specimens (due to the higher lateral size of the trenches). As expected, the microscratched surfaces exhibit the highest R_q_ and PTV values, which are within the submicron scale domain (i.e., R_q_ values are well above 100 nm and PTV values approach 1 μm). On the contrary, both the mirror-like and the electrochemically-treated surfaces show R_q_ and PTV values falling within the nanoscale. The R_q_ and PTV values of the electrochemically-treated surfaces are slightly higher than those of the mirror-like surfaces, which is consistent with the existence of an oxide porous network. Therefore, the trends in R_q_ and PTV values go in the following direction for both alloys: microscratched > nanomesh > mirror-like.

**Table 1:** Root-mean-square (RMS) roughness (R_q_) and peak-to-valley (PTV) distance determined by AFM, and contact angle values for the different surfaces.

	Ti_40_Zr_10_ Cu_38_Pd_12_			Ti-6Al-4V		
	Mirror-like	Nanomesh	Microscratched	Mirror-like	Nanomesh	Microscratched
R_q_ (nm)	3	8	141	2	11	159
PTV (nm)	28	60	776	12	80	803
Contact angle (degree)	78	84	81	89	72	71

Concerning wettability properties, contact angles between 71° and 89° were measured regardless the surface condition. Table 1 lists the static contact angle measurements, showing that both the nanomeshed and microscratched Ti-6Al-4V surfaces exhibit the lowest contact angles.

### Cell viability and cell proliferation

**Fig 3:**
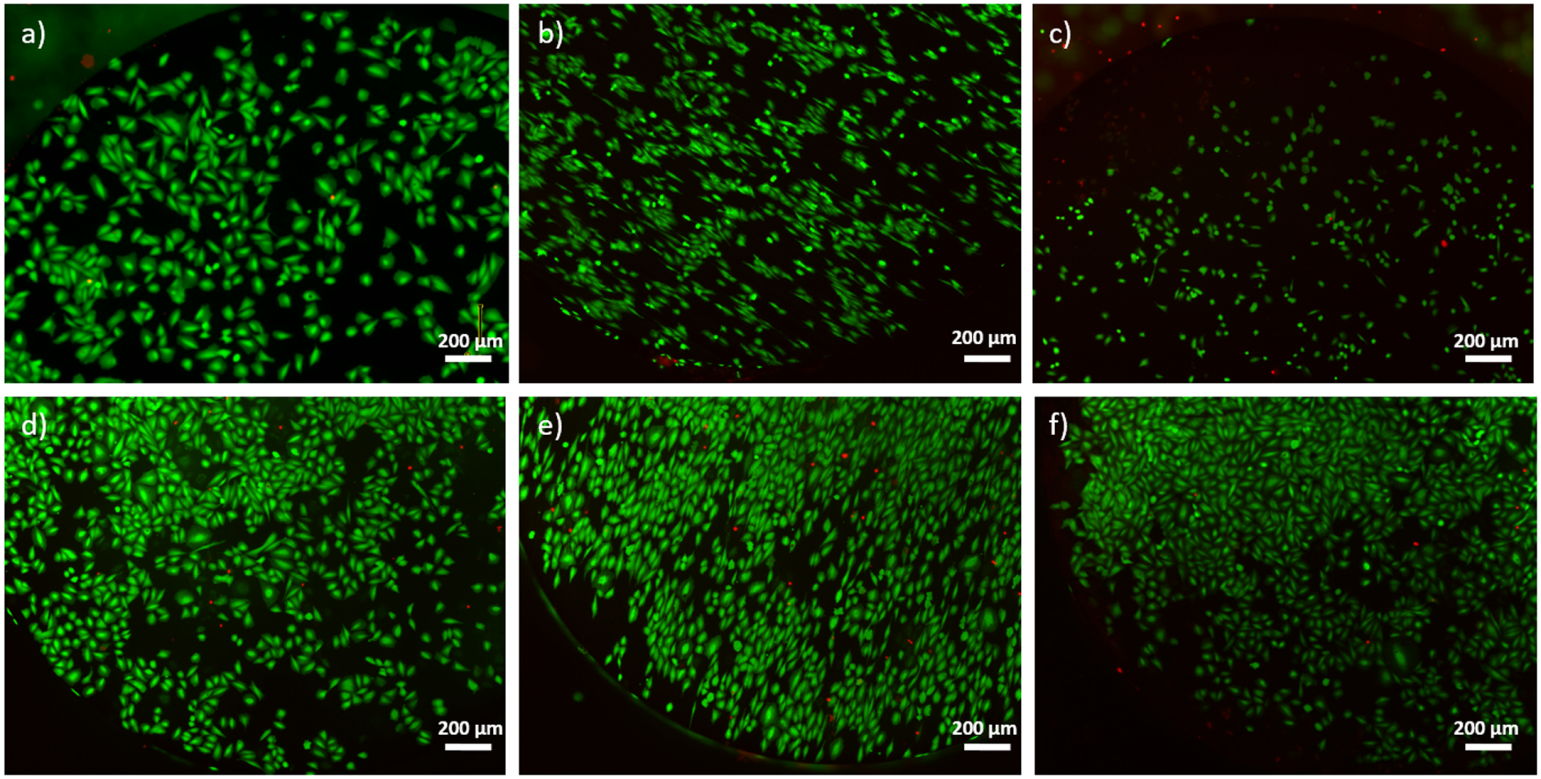
Viability of Saos-2 cells cultured onto TiZrCuPd (a-c) and Ti-6Al-4V (d-f) alloys: mirror-like (a, d), microscratched (b, e) and nanomesh (c, f). Live cells are stained in green whereas dead cells are stained in red.

Cell viability was determined by analysing Saos-2 cells grown on the surface of TiZrCuPd and Ti-6Al-4V alloys. The number of live cells detected by Live/Dead kit was higher than 96% for the three topographies studied for both alloys (Fig 3).

**Fig 4:**
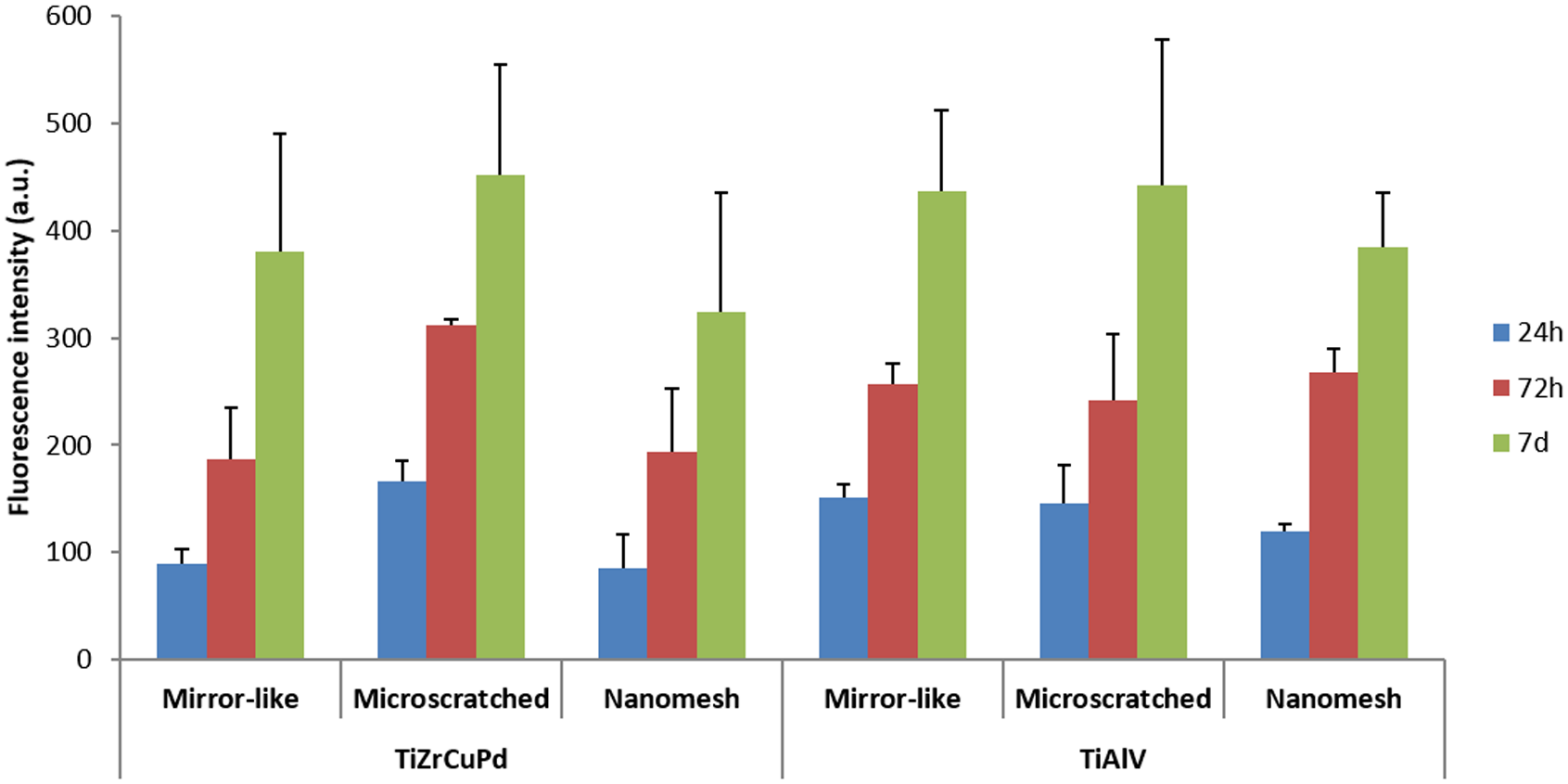
Saos-2 cells proliferation on TiZrCuPd and Ti-6Al-4V mirror-like, microscratched and nanomesh surfaces measured by Alamar Blue fluorescence at 24 h, 72 h and 7 days.

Saos-2 cells proliferation was analysed at 24 h, 72 h and at 7 days of growth on the alloy disks for the three different surface topographies of the two compositions tested (Fig 4). No significant differences between both alloys were observed for any topography at any time point.

### Cell adhesion and cell morphology

**Fig 5:**
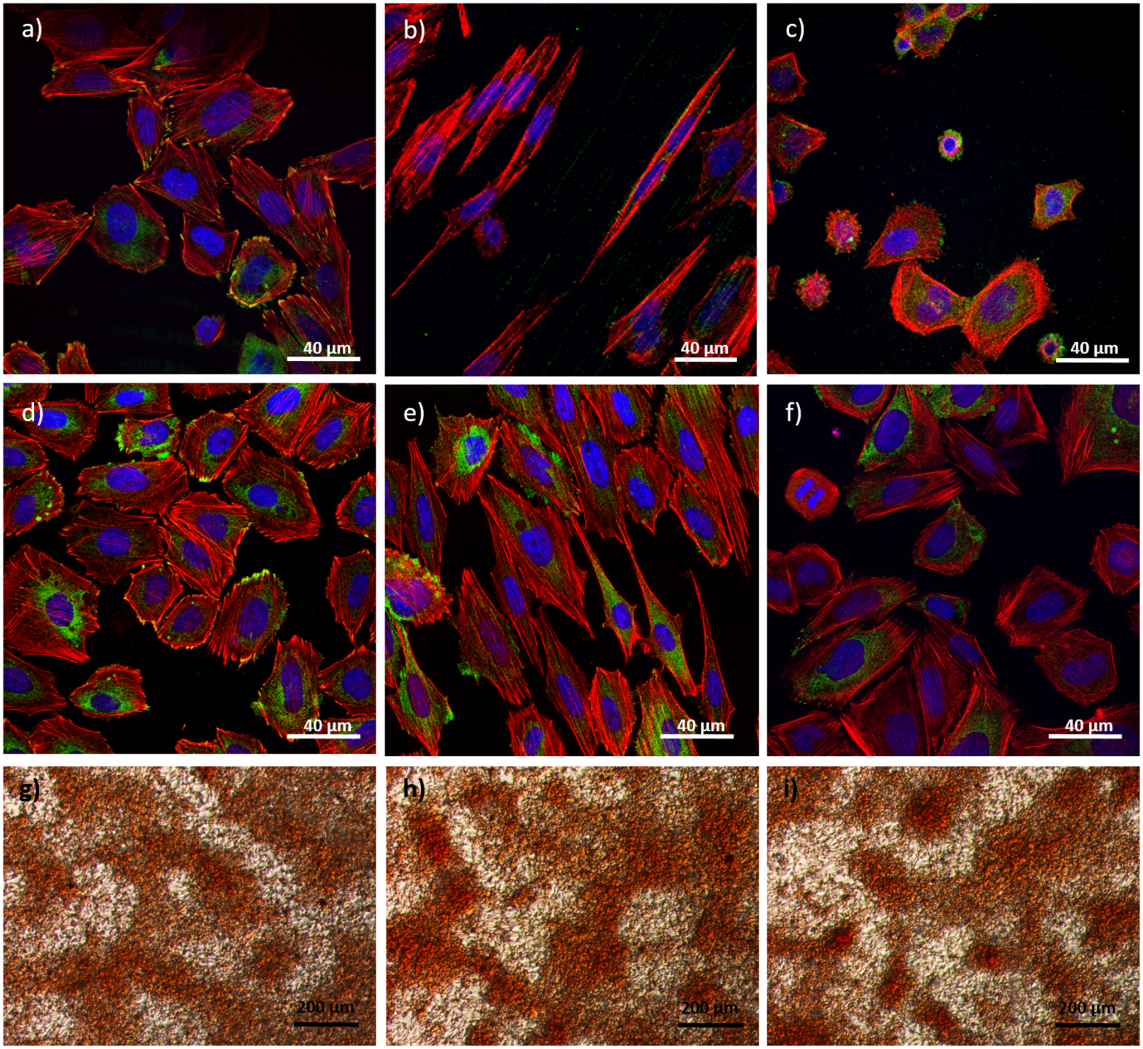
Saos-2 adhered onto the surface of TiZrCuPd (a-c) and Ti-6Al-4V (d-f) alloys: mirror-like (a, d)), microscratched (b, e)) and nanomesh (c, f)). Stress fibres (red), focal contacts (green) and nuclei (blue) can be observed. Secreted calcium deposits in the surrounding area of mirror-like (g), microscratched (h) and nanomesh (i) TiZrCuPd surface disks, detected using Alizarin Red S staining.

**Fig 6:**
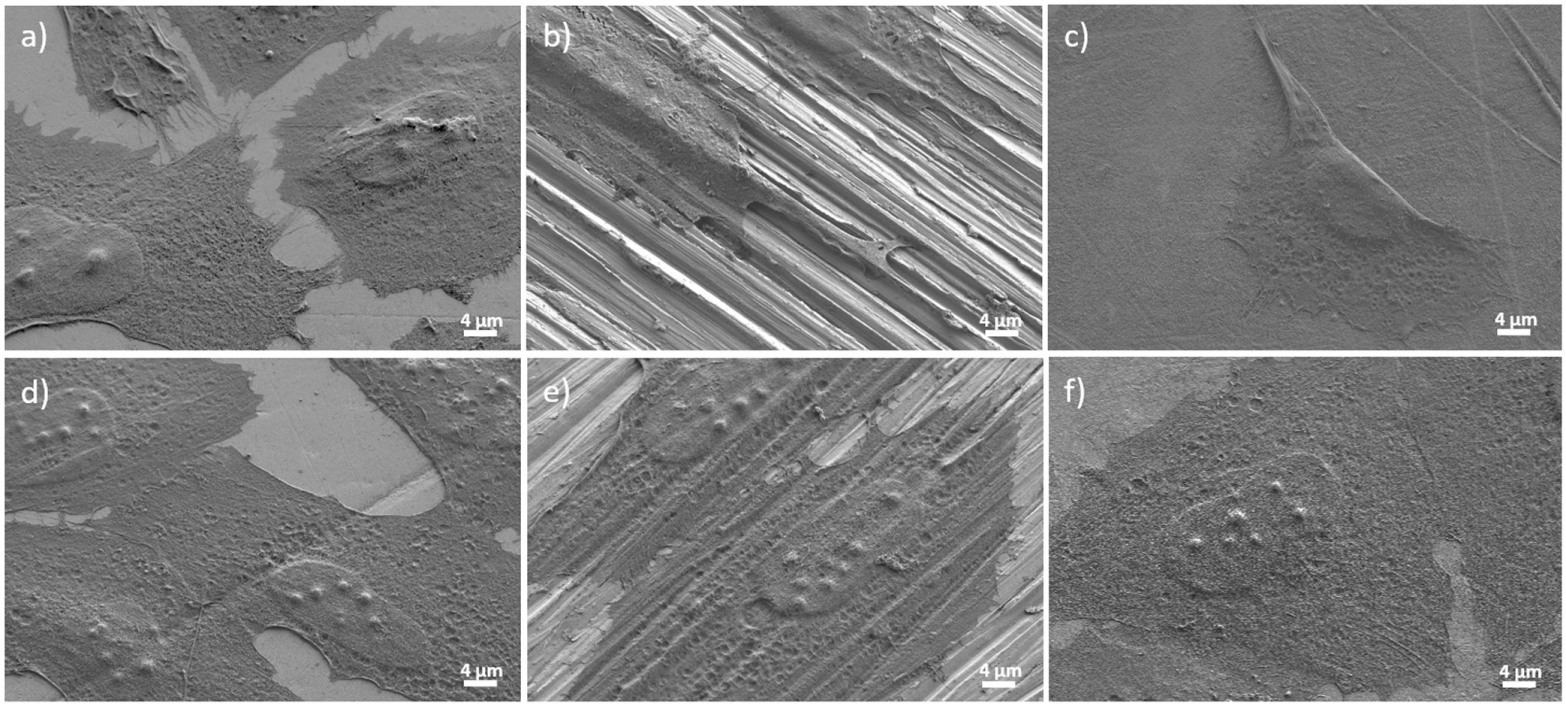
SEM images of Saos-2 cells grown on three different surface topographies of TiZrCuPd (a-c) and Ti-6Al-4V alloys (d-f): mirror-like (a, d), microscratched (b, e) and nanomesh (c, f).

Cell adhesion and cell morphology were studied after 24 h of culture using CLSM and SEM. The presence and distribution of vinculin and actin microfilaments was analysed in cells growing on the surface of the alloys with different surface topographies (Fig 5). Clear focal adhesion plaques were observed on mirror-like and nanomesh surfaces, but no recognizable focal adhesion plaques were detected on microscratched surfaces. On the other hand, actin stress fibres were normally formed in cells growing on all surfaces, although for both alloys, the orientation of cells and the arrangement of actin bundles varied as a function of the alloy surface topography. Specifically, cells growing on the microscratched surfaces showed well-defined stress fibres and a more elongated morphology compared to cells growing on mirror-like and nanomesh surfaces. In addition, cells grown on nanomesh surfaces exhibited thin and long filopodia, not usually seen in cells grown on the other surfaces. In some cells, these cytoplasm projections crossed the grooves without touching the groove floor, whereas in some other cases, the filopodia crossed the grooves touching the floor (Fig 6).

### Cell orientation

CLSM images of cells grown on the surface of the alloys were analysed to determine the cytoskeleton distribution and the cell orientation. Cell orientation was measured by the angle formed between the major cell axis and the groove axis, with 0° indicating a perfect alignment and 45° a random orientation. Stress fibres were oriented along the longitudinal axis of the microscratched surface, but no defined orientation was observed in cells grown on mirror-like and nanomesh surfaces (Fig 5). Angular measurements revealed a strong orientation of cells grown on microscratched surfaces of both alloys along the grooves with mean angles of 15.9° and 10.2° for the TiZrCuPd and TiAlV alloys, respectively. In contrast, cells grown on mirror-like or nanomesh surfaces showed a random orientation, with angles of 47° and 38°, respectively.

### Cell differentiation and ECM mineralization

**Fig 7:**
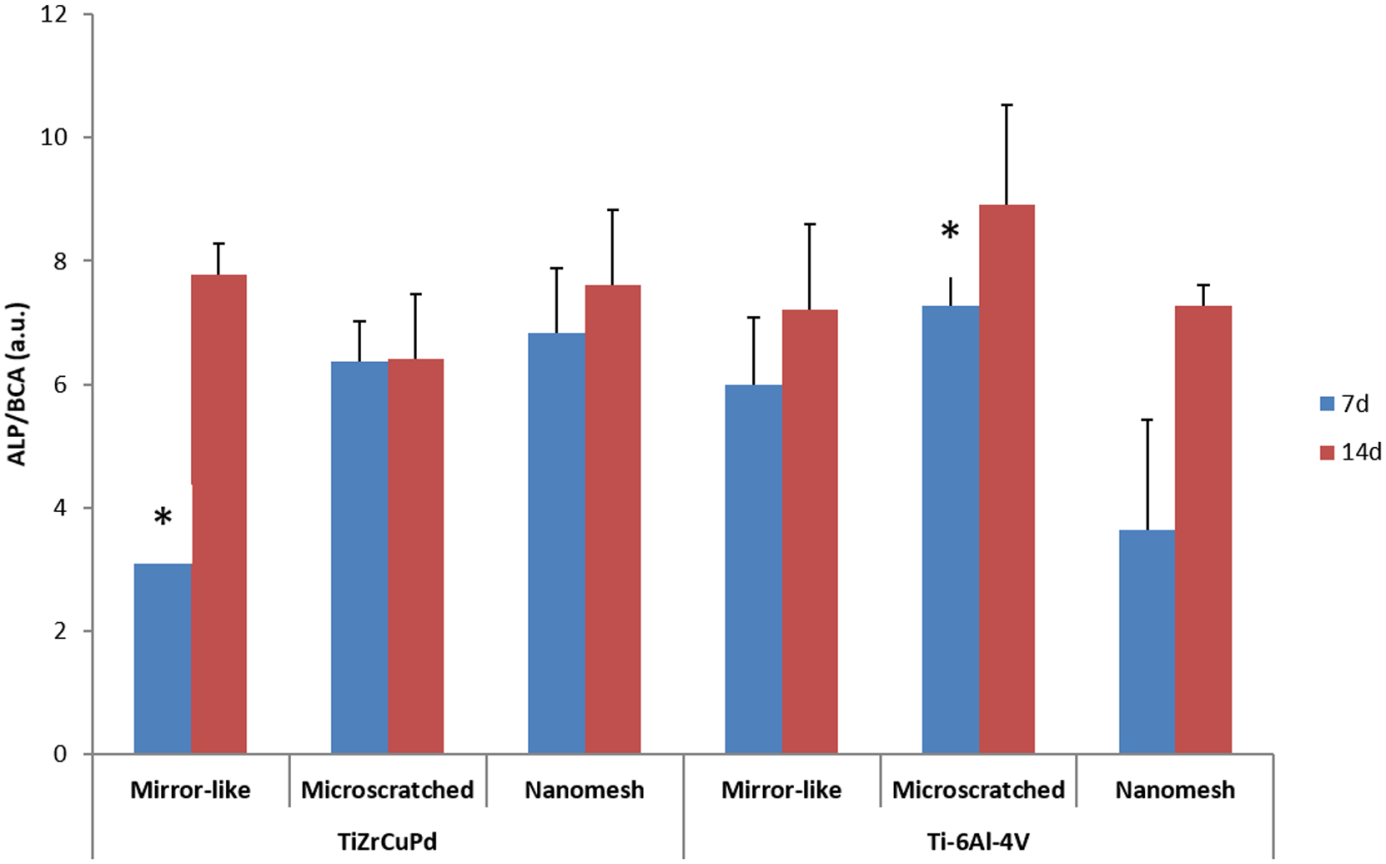
ALP activity of Saos-2 cells differentiated on three different surface topographies of TiZrCuPd and Ti-6Al-4V alloys, detected by p-nitrophenol absorbance normalized to total protein. Asterisks denote significant differences between the marked groups.

As a marker of osteoblasts differentiation, the increase of ALP activity after 7 and 14 days in culture was measured (Fig 7). No significant differences for ALP activity were observed, except for cells grown on Ti-6Al-4V microscratched surfaces when compared with cells grown on TiZrCuPd mirror-like surfaces after 7 days in culture. However, this difference disappeared after 14 days in culture, when no significant differences were observed for any alloy or surface topography.

ECM mineralization after 14 days in culture, detected as calcium phosphate nodules using Alizarin Red S stain, showed calcium nodules and similar patterns of ECM mineralization for both alloys and all topographies (data not shown).

## Discussion

Amorphous TiZrCuPd alloy shows high hardness, relatively low Young’s modulus, moderate plasticity and good corrosion resistance [23], which constitute excellent characteristics for its use in orthopaedic implants. Regarding its biocompatibility, in a previous study we reported no cytotoxic effects on mouse MC3T3-E1 preosteoblasts cultured on top of TiZrCuPd disks up to 21 days, in spite of the release of Cu ions from the alloy into the culture medium [8]. Moreover, no inflammatory cytokines secretion was observed in macrophages grown in the presence of the alloy. In order to further demonstrate its safeness for medical applications, in vitro biocompatibility analyses have now been performed with human Saos-2 osteoblast-like cells, which show similarities with primary osteoblast cells and are the best choice for osteocompatibility studies [24]. Results of cell viability, adhesion and differentiation of human Saos-2 cells cultured on top of the mirror-like TiZrCuPd alloy were similar to those previously obtained with mouse MC3T3-E1 cells [8]. In addition, no differences in biocompatibility were detected in the present study between the TiZrCuPd alloy and the commercially available Ti-6Al-4V alloy, which was used as a control biomaterial due to its known biocompatibility and its current use in medical applications [1]. Altogether, these results show that mirror-like TiZrCuPd is not cytotoxic and allows cell adhesion and differentiation on flat surfaces. The results are in agreement with other studies of Ti-based and Zr-based alloys with mirror-like surfaces [13,25,26].

To survive and proliferate, osteoblasts need to adhere to a surface through focal contacts. In this sense, it is known that the surface topography of an alloy can influence cell adhesion, and thus cell proliferation and differentiation [11,27,28]. It has been reported that nanorough surfaces provide better cell proliferation and differentiation than smooth ones [17], even though other authors reported that better results are obtained with microrough surfaces [28]. A drawback for standardizing the knowledge on the importance of topography on cell behaviour is the lack of consensus for surface topography characterization [29]. Because there are not many studies comparing the biological effect of different topographies of an alloy in the same cell line, we focused the present study on comparing the biological effect, in Saos-2 cells, of three different topographies of the TiZrCuPd alloy: mirror-like, microscratched and nanomesh. As a control we used the commercial Ti-6Al-4V alloy, treated in the same fashion to obtain the same three topographies.

Electrochemical polarization and directional manual scratching was used to change the surface topography and the physicochemical properties of the alloys. In the present study, anodic oxidation generated an interconnected hierarchical porous structure by potentiodynamic polarization in 5M NaOH solution at 25°C. Coral-like topographies have been reported to form upon soaking Ti in hot NaOH (70°C) for 24h [30]. Under these conditions, chemical etching of Ti takes place. Here, a spongy morphology was developed by polarizing the material in NaOH at RT. A similar procedure has been previously applied to steels, by scanning the potential from +1.6 to +2.6 V in 50% NaOH at temperatures ranging from 30°C to 70°C [31]. EDX studies showed that the nanomesh layer of the TiZrCuPd alloy was rich in Ti and Cu oxides, and that of the Ti-6Al-4V alloy was rich in Ti oxides. Other authors have described that electrochemical anodization of Ti-based alloys produces TiO_2_ structures on their surfaces, such as TiO_2_ nanotubes [20] or a TiO_2_ nanomesh [21]. This TiO_2_ surface layer plays an important role acting as an inhibitor of metallic ions release and increasing the corrosion resistance of the alloy [21,32]. Moreover, TiO_2_ has been considered biocompatible and an enhancer of the biological response [21,32,33]. Manual disk scratching created parallel trenches without changing the surface composition. R_q_ values of scratched surfaces were in the submicron microscale domain whereas those of the electrochemical-treated and mirror-like surfaces were at the nanoscale, showing higher values on the electrochemical-treated surfaces. Contact angle measurements of all surfaces were below 90°, which is the threshold for hydrophobicity. Therefore, the surfaces can be regarded as slightly polar. The value for the Ti-6Al-4V alloy with mirror-like finish is comparable to the previously reported contact angle for metal oxides (considering that a natural TiO_2_ passivation layer forms on the surface) [32].

It has been described that when grown on Ti-6Al-4V microrough surfaces, primary human osteoblasts show a more differentiated phenotype than mesenchymal stem cells [28]. Moreover, Li et al. indicated that Ti and Zr BMG microroughness enhance MG63 osteosarcoma cells attachment, proliferation and differentiation [15]. It has also been described that differentiation of human primary osteoblasts is enhanced by nanostructured superimposed onto micro-rough Ti-6Al-4V surfaces [34]. Similarly, Deng et al. concluded that nanostructured Ti is beneficial for MG63 osteosarcoma cells adhesion, viability and differentiation [17]. In contrast with all these previous reports, our study with Saos-2 cell line shows no significant differences in terms of cell viability, proliferation and differentiation among the three types of evaluated surfaces neither for the Ti-6Al-4V alloy, nor for the TiZrCuPd. The surface-modified-samples (microscratched and nanomesh) were as good as mirror-like ones for cell adhesion, proliferation and differentiation indicating that the three types of surfaces, can potentially be used in orthopaedic implants.

A previously described orientation effect has also observed on microscratched surfaces. It has been reported that cells are able to recognize the topography from few nanometers to hundred microns [29], and that cell morphology and orientation can change depending on topography. When topography presents specific patterns, such as grooves or ridges, cell orientation can be measured by the angle formed between the major cell axis and the groove axis. Some authors have described that cells can be aligned along defined substrates and, in most of the cases, orientation is obvious due to the formation of filopodia at opposite ends of the cell [35]. In our work, microscratched surfaces present grooves with a RMS of approximately 150 nm and the maximum PTV value is 789 nm. Measurements of cell orientation on microscratched surfaces showed a mean angle of 15.9°, indicating a good alignment with topography grooves. Indeed, most of the cells analysed had cytoplasm projections and actin microfilaments aligned along the grooves. In any case, taking into account our biocompatibility results, differences in cell orientation do not seem to influence Saos-2 osteoblast viability, proliferation or differentiation.

## Conclusion

Our results show that Ti_40_Zr_10_ Cu_38_Pd_12_ BMG can be considered an excellent candidate to be used for orthopaedic implants. Moreover, we have demonstrated that electrochemical treatment generates a porous structure (nanomesh) rich in oxides on both Ti-6Al-4V and Ti_40_Zr_10_ Cu_38_Pd_12_ alloys, which improve corrosion resistance. Regarding biological studies, surface modifications do not show any impact on proliferation, adhesion and differentiation of human Saos-2 cells. A directional attachment of cells is observed on surfaces featuring parallel trenches created by manual scratching. The results provide an interesting contribution to the biological effect of surface modifications and their potential use to improve osseointegration.

## Supporting Information

S1 Fig SEM image of the nanomesh layer formed by electrochemical treatment on Ti-6Al-4V alloy. (TIF)

S2 Fig Potentiodynamic polarization curve in logarithmic scale of Ti_40_Zr_10_Cu_38_Pd_12_ alloy in 5 M NaOH at 25°C (scan rate 0.5 mV s^-1^). (TIF)
