# Supplementary figures and images for: Effect of Surface Modifications of Ti40Zr10Cu38Pd12 Bulk Metallic Glass and Ti-6Al-4V Alloy on Human Osteoblasts In Vitro Biocompatibility
Source: PLoS One. 2016 May 31;11(5):e0156644. doi: 10.1371/journal.pone.0156644 (PMC4887090; doi:10.1371/journal.pone.0156644)

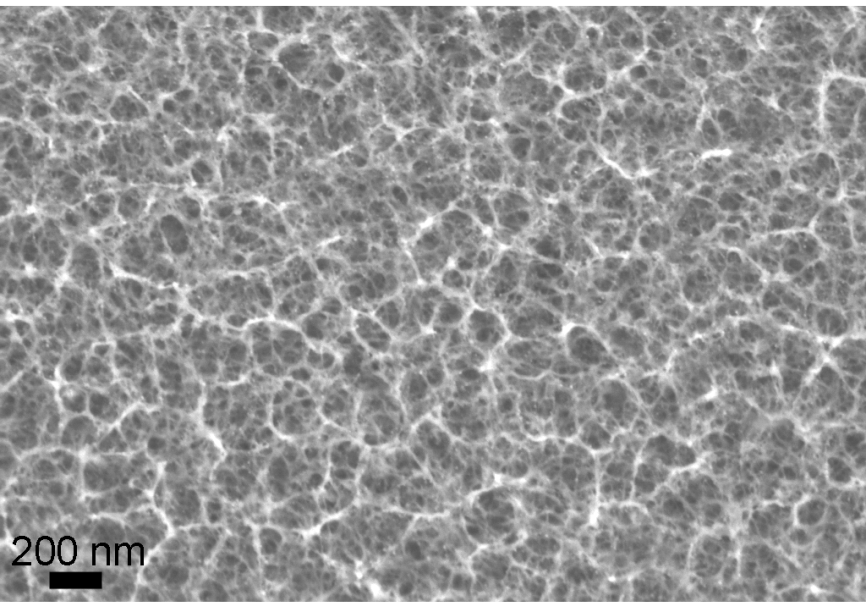

Supplement: S1 Fig — (TIF) [file pone.0156644.s001.tif]
